# Global Trends in Polycystic Ovary Syndrome Burden, 1990–2021: Insights from the Global Burden of Disease Study

**DOI:** 10.1177/26884844251395123

**Published:** 2025-11-11

**Authors:** YunLan Wang, DongYi Shen, Chan Zhu, Wei Sun, Yun Qian, Hong Yang

**Affiliations:** ^1^ Department of Gynecology, Shanghai Municipal Hospital of Traditional Chinese Medicine, Shanghai University of Traditional Chinese Medicine, Shanghai, China.; ^2^ Department of Gynaecology and Obstetrics, Shuguang Hospital Affiliated to Shanghai University of Traditional Chinese Medicine, Shanghai, China.; ^3^ Department of Traditional Chinese Medicine, West China Second University Hospital, Sichuan University, Chengdu (Sichuan), China.; ^4^ Key Laboratory of Birth Defects and Related Gynecological Diseases of Women and Children, Sichuan University, Chengdu (Sichuan), China.

**Keywords:** Global Burden of Disease, polycystic ovary syndrome, age–period–cohort, health inequality analysis, frontier analysis, epidemiology

## Abstract

**Objective:**

Utilizing the 2021 Global Burden of Disease (GBD) data, this study assessed the global epidemiological landscape of polycystic ovary syndrome (PCOS), offering an empirical foundation for PCOS-related health service planning.

**Methods:**

Data from the GBD 2021 database were extracted to examine the global distribution and trends of PCOS. The metrics included total case counts, prevalence, disability-adjusted life years, age-standardized prevalence rate (ASPR), and estimated annual percentage change (EAPC). Analyses incorporated age–period–cohort modeling and frontier efficiency assessments.

**Results:**

Between 1990 and 2021, the global ASPR of PCOS exhibited a marked upward trajectory. Significant regional heterogeneity was observed; Southeast, East, and South Asia registered the steepest ASPR growth, whereas tropical Latin America and high-income Asia Pacific displayed relatively stable trends. At the national level, countries such as Equatorial Guinea, Maldives, and Myanmar recorded ASPR increases exceeding 100%, in contrast to reductions seen in high-income nations including Italy. Age-specific patterns revealed that women aged 20–44 years consistently bore the highest burden. The age–period–cohort analysis indicated heightened risk in more recent birth cohorts from low- and middle-socioeconomic development index (SDI) regions, coupled with persistent health disparities despite some narrowing in socioeconomic gaps, as reflected by a decline in concentration indices from 0.24 to 0.1. The frontier analysis identified high-SDI countries, including the United States and Japan, as performing suboptimally in mitigating the PCOS burden relative to their resource capacity.

**Conclusion:**

The global burden of PCOS has intensified over the past three decades, with a disproportionate impact in low- and middle-SDI regions. The syndrome predominantly affects women of reproductive age, especially those between 20 and 39 years of age. Although socioeconomic inequalities have lessened to some extent, disparities remain significant, and resource-rich nations continue to underperform in addressing the disease burden. Comprehensive strategies emphasizing timely diagnosis, equitable health care access, and lifestyle interventions are imperative to address this escalating global health concern.

## Introduction

Polycystic ovary syndrome (PCOS), the most common endocrine disorder in reproductive-aged women globally, is currently defined according to several overlapping but distinct sets of criteria—including those established by the National Institutes of Health (NIH) and the Rotterdam consensus—which may lead to differing prevalence estimates and complicate efforts to obtain a consistent global burden assessment.^1^ The data used in this study mainly refer to the diagnostic criteria of the NIH, because the official Global Burden of Disease (GBD) website states that its definition of PCOS is as follows: PCOS is defined according to the NIH criteria, which include chronic anovulation and hyperandrogenism (established by hormone measurements or clinical findings) in women in whom secondary causes have been excluded. It means that assessment protocols must exclude alternative sources of androgen excess and identify coexisting risk factors for endometrial cancer, mood disorders, obstructive sleep apnea, diabetes, and cardiovascular disease.^2,3^ Nonetheless, PCOS frequently coexists with these conditions, particularly due to its strong association with insulin resistance, dyslipidemia, and obesity, thereby substantially elevating the risks of type 2 diabetes, gestational diabetes, pregnancy complications, venous thromboembolism, cerebrovascular and cardiovascular events, and endometrial cancer.^4,5^ Furthermore, genetic predispositions have been implicated in its pathogenesis.^6^ In recent decades, the global burden of PCOS has escalated, possibly fueled by rising obesity rates and exposure to environmental endocrine-disrupting chemicals such as bisphenol A,^7^ rendering it an increasingly significant public health concern affecting women’s overall well-being worldwide.^8^ In addition, a previous study by Gao et al. (2023) also examined the global PCOS burden from 1990 to 2019.^9^ Our study extends this by including data through 2021, incorporating age–period–cohort (APC) and frontier analyses, and explicitly addressing socioeconomic inequities—thereby providing a more updated and multidimensional assessment.

Based on data from the GBD 2021 database and standardized by the United Nations criteria, this study delineates the global epidemiological profile of PCOS in women, examining temporal trends in prevalence across countries and regions and assessing its associations with age, period, and birth cohort, because we consider that changes in factors, such as changes in the socioeconomic environment, alterations in the dietary structure, and changes in lifestyle, may affect the risk and detection of PCOS across different periods and birth cohorts. That is to say, we want to test the following hypothesis: the temporal trend of PCOS is affected by age, period, and birth cohort in different ways, and these effects vary under different levels of socioeconomic development, we conducted an APC analysis. In addition, previous studies have also shown that socioeconomic status may influence both the risk and diagnosis of PCOS.^10,11^ The socioeconomic development index (SDI) is a composite indicator used in sociology to comprehensively measure the social, economic, and population development levels of a region or country. By quantifying and weighting multiple key factors, such as economic development level, education level, population density, urbanization level, and other social factors, it provides a numerical value that reflects the overall social development of a certain region.^12^ Therefore, it is necessary to incorporate the SDI to analyze the impact of development levels in different regions and countries on the epidemiology of PCOS. Moreover, although PCOS is primarily identified in reproductive-aged women, we have noticed that there are still patients with PCOS among non-fertile-age women. Even if they have no fertility needs, they still need to receive treatment to prevent long-term adverse outcomes. Thus, understanding how age-specific prevalence varies within different groups remains essential for informing targeted interventions.

By elucidating these patterns, this study seeks to support the development of region-specific public health strategies, including tailored lifestyle interventions and health care services, particularly in high-risk and resource-limited settings. The findings aim to strengthen the evidence base for future efforts aimed at mitigating the burden of PCOS globally.

## Materials and Methods

### Data source

PCOS data spanning 1990 to 2021 were retrieved from the Global Health Data Exchange (GHDx) query tool (https://vizhub.healthdata.org/gbd-results/), which systematically compiles information on the global burden of 369 diseases and injuries. The annually updated datasets reflect temporal shifts in global health metrics and support projections for future health care demands.^12,13^

The GBD 2021 study incorporates the SDI, as introduced earlier, which is a socioeconomic metric that can be used to quantify it’s influence on health outcomes, with scores ranging from 0 to 1. SDI levels are categorized as low, low-middle, middle, high-middle, and high. A score of 0 indicates minimal education, lowest income levels, and highest fertility rates, whereas a score of 1 denotes the converse.^12^

The GBD 2021 methodology adheres to the Guidelines for Accurate and Transparent Health Estimates Reporting (GATHER) standards. Given the public accessibility of the dataset, ethical approval was not required.

All analyses were conducted using R version 4.4.1 (R Foundation, Vienna, Austria).

### Statistical analysis

#### Trend analysis

The estimated annual percentage change (EAPC) in the age-standardized prevalence rate (ASPR) of PCOS was assessed globally and across regions using regression models to quantify temporal shifts in prevalence over the study period. The EAPC was calculated by fitting a linear regression to the natural logarithm of the ASPR, with the calendar year as the independent variable. The formula used was as follows: *ln(ASPR) = α + β × year + ε*, where β represents the annual change, and the EAPC was computed as 100 × *(e^β* − 1). If the EAPC is positive, it indicates that the ASPR shows an annual upward trend over the years; if it is negative, it indicates an average annual downward trend; and if it is close to 0, it means that the trend is stable. This method allows for the quantification of the average annual rate of the change in prevalence, adjusted for age distribution differences over time and across populations.

#### Regional and national heterogeneity analyses

Country-level prevalence counts and ASPRs were analyzed independently. Geospatial visualization enabled the classification of countries based on the magnitude of prevalence variation, supporting comparative regional assessments.

#### Age-stratified analysis of global and five SDI regions in PCOS prevalence

PCOS prevalence was evaluated in discrete age intervals from 10–14 to 50–54 years across five SDI regions. This stratification enabled the identification of age-dependent patterns in the disease burden and elucidated its distribution across distinct reproductive stages.

#### APC analysis

To test the following hypothesis: the temporal trend of the PCOS is affected by age, period, and birth cohort in different ways, and these effects vary under different levels of socioeconomic development, we conducted an APC analysis. The model was implemented using the APC package in R, which employs intrinsic estimator techniques to address the identifiability issue (the linear dependence among age, period, and cohort). The analysis produced coefficient plots for age, period, and cohort effects, facilitating the assessment of life-course risk, temporal trends, and generational shifts in PCOS prevalence. This approach enabled the comprehensive interpretation of time-specific and generational influences on disease trends.

#### Health inequality analysis

The slope index of inequality (SII) and the concentration index (CI) were employed to quantify socioeconomic disparities in PCOS prevalence across 204 countries and regions. The SII was derived *via* a national rate regression model anchored to the SDI rank-based scale, using the midpoint of the cumulative population (ordered by the SDI) as the reference. To mitigate heteroscedasticity, weighted regression modeling was applied. The CI was determined by calculating the area between the Lorenz curve and the line of equality, with the curve constructed from the cumulative population distribution and the corresponding SDI rankings.^14^ These metrics independently capture absolute and relative inequality, with greater deviation from zero denoting intensified disparity. Positive SII or CI values signify a concentration of the burden in higher SDI settings, whereas negative values indicate a disproportionate burden in lower SDI countries.

#### Frontier analysis

A frontier analysis was conducted to assess optimal PCOS burden management under varying SDI conditions. This method identifies a “best practice” frontier—a curve representing the most favorable (lowest achievable) ASPR for a given SDI—based on countries with the most efficient health systems. Countries were then stratified based on their deviation from this frontier fit line, enabling the identification of those achieving efficient disease burden control and those requiring strategic improvement. The analysis helps pinpoint nations that outperform or underperform relative to their socioeconomic peers, thereby informing targeted policy interventions.

## Results

### Global and regional burden of PCOS (1990–2021)

**FIG. 1.**
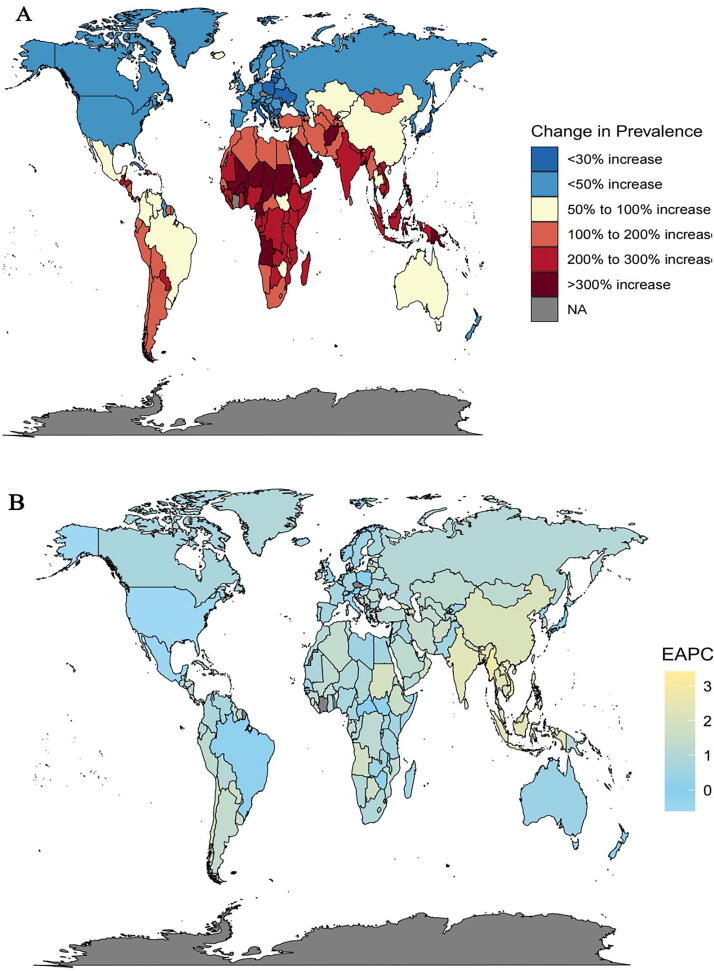
Global temporal dynamics in the PCOS burden. (**A)** Percentage change in prevalence across 204 countries between 1990 and 2021. **(B)** EAPC in prevalent rates across 204 countries from 1990 to 2021. PCOS, polycystic ovary syndrome; EAPC, estimated annual percentage change.

Over the period from 1990 to 2021, the global burden of PCOS demonstrated a marked upward trajectory. According to Supplementary Table S1, the ASPR rose from 1372.77 per 10,000 individuals (95% uncertainty interval [UI]: 984.64–1891.60) in 1990 to 1757.83 per 10,000 individuals (95% UI: 1253.36–2421.26) in 2021. Supplementary Table S2 detailed the percentage change in the ASPR across this time frame. Among the 21 assessed regions, Southeast Asia (86.73%, 95% UI: 73.71%–100.3%), East Asia (83.12%, 95% UI: 71.77%–94.74%), and South Asia (76.71%, 95% UI: 66.1%–90.73%) recorded the most pronounced increases. In contrast, tropical Latin America (9.19%, 95% UI: 4.2%–14.73%), Central Latin America (10.09%, 95% UI: 6.11%–14.66%), and high-income Asia Pacific (10.26%, 95% UI: 4.72%–16.33%) experienced comparatively limited growth. In addition, Figure 1 highlighted that the relative increase in prevalent PCOS cases surpassed 300% in East Africa and the Middle East over the same period. The EAPC in the ASPR further emphasized regional disparities, with the most notable shifts concentrated in East and Southeast Asia, whereas Europe, North America, and Australia displayed relatively stable patterns.

### National heterogeneity in PCOS burden

Figure 1 and Supplementary Table S2 presented the percentage change in prevalence counts between 1990 and 2021. Based on the data from the GBD database, the ASPR of PCOS in 2021 ranged from 196.09 (95% UI: 127.73–299.07) in North Macedonia to 980.60 (95% UI: 674.56–1378.28) in the Democratic People’s Republic of Korea, whereas in 1990, the values spanned from 145.74 (95% UI: 96.31–222.52) in Albania to 919.13 (95% UI: 637.00–1310.80) in South Africa. The derived percentage changes in the ASPR identified Equatorial Guinea (134.66%, 95% UI: 107.2%–178.4%), Maldives (133.15%, 95% UI: 103.85–168), Myanmar (106.34%, 95% UI: 79.02%–135.47%), and Viet Nam (101.32%, 95% UI: 76.73%–127.29%) as exhibiting the most substantial increases. In contrast, high-income countries such as Italy (−5.21%, 95% UI: −11.86–0.32) demonstrated declining trends, whereas Mexico (0.82%, 95% UI: −4.93–6.73) and New Zealand (1.3%, 95% UI: −11.98%–12.7%) reported relatively minor increases.

### Age-stratified trends of global and five SDI regions in PCOS prevalence

**FIG. 2.**
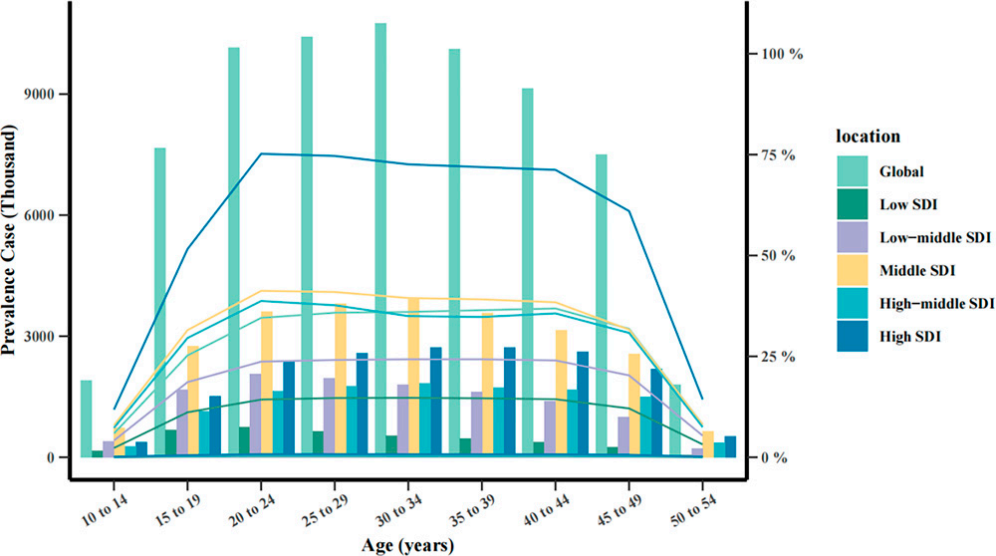
Age-specific prevalence counts and annual rates per 10,000 women in 2021.

Figure 2 illustrated the age-specific prevalence of PCOS cases in 2021 globally and across the five SDI regions, covering age groups from 10–14 to 50–54 years in 5-year intervals. The largest number of cases globally was observed among women aged 30–34 years (10,763,002; 95% UI: 7,711,792–14,968,295), followed closely by those aged 25–29 (10,422,751; 95% UI: 7,483,904–14,456,356), 20–24 (10,146,098; 95% UI: 7,311,868–13,931,505), and 35–39 (10,125,830; 95% UI: 7,189,683–14,078,426) years. Case numbers subsequently declined in older age brackets: 40–44 (9,147,146; 95% UI: 6,544,589–12,706,765) and 45–49 (7,499,319; 95% UI: 5,414,709–10,384,919) years, and younger cohorts: 15–19 (7,663,403; 95% UI: 5,183,309–10,971,947) and 10–14 (1,900,415; 95% UI: 1,006,274–3,100,290) years. The lowest prevalence was recorded in women aged 50–54 years (1,805,283; 95% UI: 1,249,484–2,521,080). Among the SDI regions, middle-SDI regions reported the highest absolute number of cases, whereas the lowest burden was recorded in low-SDI regions, signifying a clear gradient in the prevalence distribution linked to the sociodemographic context. This variation indicates an unequal disease burden across regions, with middle-SDI areas disproportionately affected within the assessed age range. Furthermore, Figure 2 showed that the highest annual prevalence rate in 2021 was detected in high-SDI regions. Prevalence increased with age, rising from 11.81% (95% UI: 5.93%–19.41%) in the 10–14-year group to a peak of 75.20% (95% UI: 55.84%–101.53%) in the 20–24-year group. The rate remained relatively stable until the 40–44-year group (71.22%; 95% UI: 52.58%–98.67%) before declining substantially in older cohorts. This trend remained consistent across the global dataset and all five SDI regions. Low-SDI regions persistently exhibited the lowest prevalence rates. Such disparities may reflect differences in health care infrastructure, diagnostic practices, environmental exposures, and lifestyle patterns across SDI strata.

### APC effects on PCOS prevalence

**FIG. 3.**
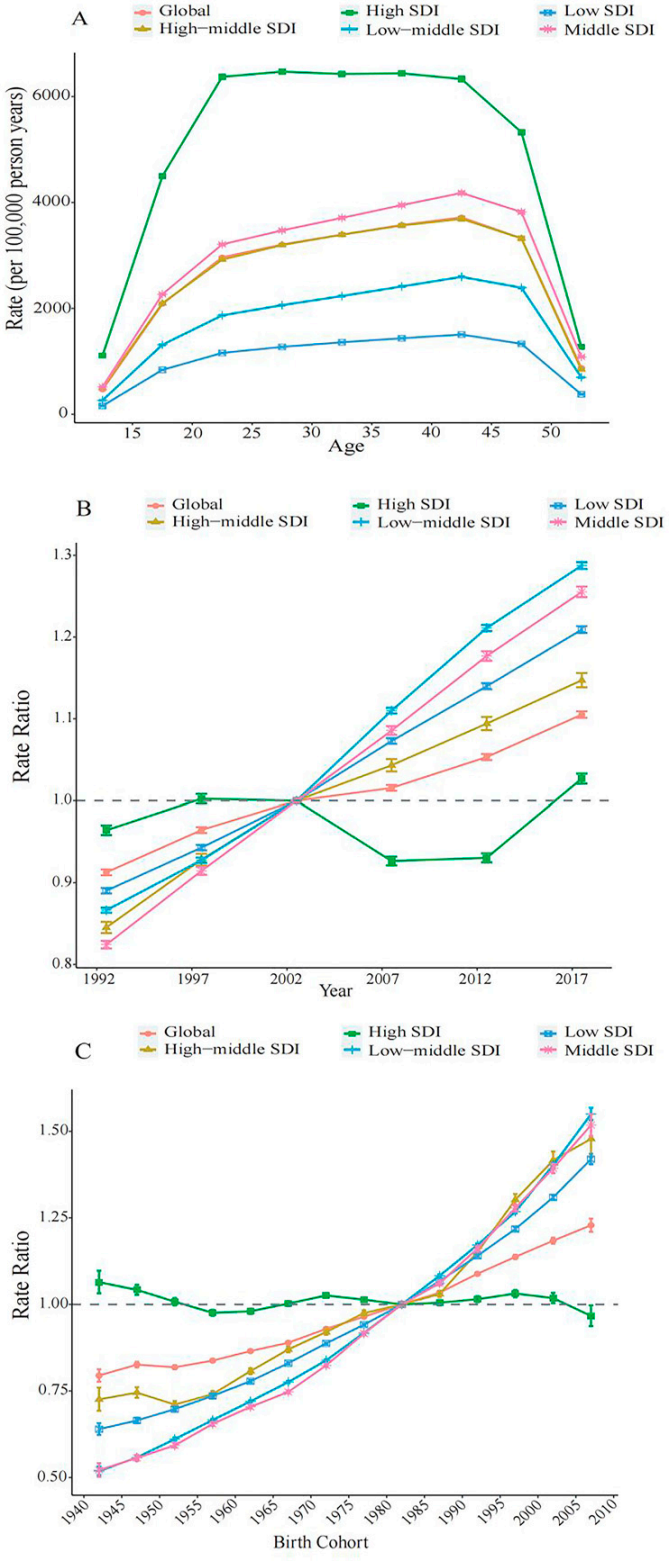
Parameter estimates of **(A)** age effects, **(B)** period effects, and **(C)** cohort effects on the prevalence rate of PCOS in global and five SDI quintiles from 1990 to 2021. SDI, socioeconomic development index.

The APC analysis revealed stratified risk dynamics across age, period, and cohort dimensions (Fig. 3). The age effect indicated a rising prevalence rate beginning at approximately 15 years, peaking between 30 and 40 years, followed by a gradual decline with advancing age. Period effects displayed heterogeneity across SDI levels. In high-SDI regions, rate ratios began declining post-2002, decreasing from approximately 1.1 to 0.9 by 2017. Conversely, an upward trend was observed in low-middle and low-SDI regions during the same time frame; rate ratios increased from approximately 0.9 to 1.1 in low-middle-SDI areas and from approximately 0.8 to 1.0 in low-SDI regions. Cohort effects further reflected regional disparities. Among later birth cohorts, particularly those born after 1990, populations in low-SDI regions exhibited consistently higher rate ratios than their high-SDI counterparts. Specifically, the rate ratio in low-SDI regions surpassed that in high-SDI regions by approximately 0.25, suggesting elevated relative risks among younger cohorts in socioeconomically disadvantaged settings.

### Health inequality analysis

**FIG. 4.**
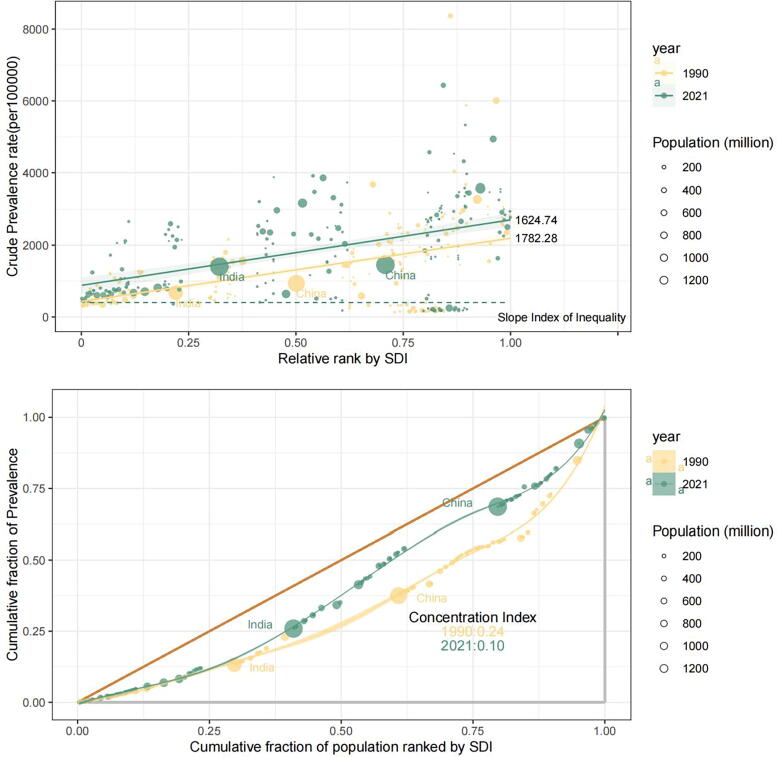
SDI-related health inequality regression and concentration curves for the prevalence of PCOS worldwide, 1990 and 2021.

Building on the association between prevalence rates and the SDI, further evaluation of health disparities was performed using the data illustrated in Figure 4. From 1990 to 2021, the ASPR slope index for PCOS declined modestly from 1782.28 to 1624.74, accompanied by a reduction in the CI from 0.24 to 0.10. The SII, indicating disparities in prevalence across SDI-stratified regions, demonstrated a marked decline over this period. The initially stronger gradient observed in 1990, signifying a tighter correlation between higher SDI and elevated prevalence, had attenuated by 2021. Similarly, the CI, quantifying inequality in the prevalence distribution across SDI-ranked populations, reflected a significant contraction—from 0.24 to 0.10—implying a gradual reduction in disparity. A comparative analysis of China and India further supported this trajectory. In 1990, divergence in the prevalence distribution between the two countries was more apparent, as suggested by the CI, whereas by 2021, this disparity had diminished, reinforcing the overarching pattern of narrowing health inequities in relation to SDI-linked prevalence.

### Frontier analysis of the association between ASPR and SDIs

**FIG. 5.**
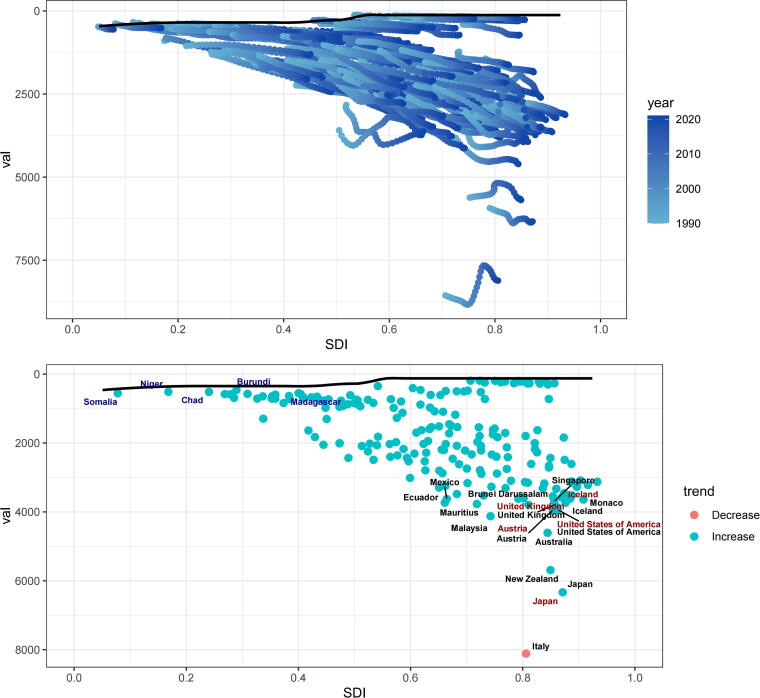
Slope indexes and concentration indexes for PCOS prevalence from 1990 to 2021 worldwide.

To identify the optimal conditions under which countries may effectively manage the PCOS burden relative to annually adjusted SDI levels, a frontier analysis was conducted (Fig. 5). Within the lower SDI group, five countries—Somalia, Niger, Chad, Burundi, and Madagascar—were positioned closest to the frontier fit line based on ASR prevalence and are denoted in blue. Their proximity suggests that, despite limited resources, their ASR prevalence aligns closely with the optimal trajectory delineated by the frontier model. In contrast, five high-SDI countries—United Kingdom, Iceland, Austria, United States, and Japan—were the most distant from the frontier line and are highlighted in red, reflecting a greater deviation from the expected performance relative to their development level. In addition, across all SDI strata, 15 countries—including the above five along with Ecuador, Mexico, Brunei Darussalam, Singapore, Monaco, Mauritius, Malaysia, Australia, New Zealand, and Italy—exhibited the greatest divergence from the frontier benchmark and are marked in black.

## Discussion

The longitudinal assessment of the PCOS burden from 1990 to 2021 delineates significant epidemiological trajectories and health inequities warranting immediate intervention. The analysis indicates a marked escalation in the global PCOS prevalence, characterized by substantial variation across geographic regions, age cohorts, and socioeconomic contexts. These disparities correlate closely with shifts in environmental exposures, demographic transitions, and health care accessibility, yielding policy-relevant implications.

A global 28.1% rise in the ASPR of PCOS parallels the increasing trends in obesity and metabolic syndrome incidence worldwide.^15^ The most significant surges occurred in Southeast Asia (86.73%) and East Asia (83.12%), probably influenced by the accelerated urbanization and adoption of energy-dense, processed diets.^16–19^ In contrast, tropical Latin America registered only a 9.19% rise, potentially attributable to an earlier plateau in obesity prevalence or underdiagnosis linked to fragmented health care infrastructure.^20–22^ The >300% escalation in East Africa and the Middle East reflects the compounded effects of nutritional transition and constrained reproductive health care access, where <30% of affected women receive timely PCOS diagnoses.^23,24^

Marked national disparities reflect the influence of socioeconomic variables. Equatorial Guinea experienced a 134.66% rise in the ASPR, contrasting sharply with a 5.21% decline in Italy. In the case of Equatorial Guinea, since the start of the 21st century, coupled with increased government investment in medical resources and support from international organizations, its diagnostic capabilities have improved, and disease awareness has been enhanced. These developments have likely facilitated the better detection of existing cases and the concentrated reporting of a large number of previously undiagnosed cases that had gone undetected due to past equipment or technical limitations. Consequently, this may lead to a significant increase in the reported prevalence.^25,26^ Conversely, the reduction in Italy aligns with early diagnostic access under universal health coverage, improvements in health status facilitated by adherence to the Mediterranean diet, and the implementation of structured public health strategies, including health education initiatives and investment in physical activity infrastructure.^27–30^ A geospatial analysis identified 80% of low- and middle-income countries (LMICs) within the “extreme growth” category (200%–300% increase), corroborating existing evidence on the associations between PCOS prevalence and environmental exposures such as air pollution and endocrine-disrupting agents in industrializing settings.^31,32^ The PCOS data collected from the GBD database, based on the NIH criteria, may reflect both the observed differences between countries based on the actual disease burden and the differences in medical diagnostic capability. High-SDI countries may have more sophisticated health care systems and may identify cases with milder symptoms, such as irregular menstruation or acne, more effectively, diagnosing more cases of PCOS that meet the NIH criteria, which may be closer to the actual disease burden. In contrast, low-SDI countries may only diagnose more severe cases based on infertility or hirsutism, leading to underdiagnosis and an underestimation of the true burden of PCOS. The low prevalence rates shown in low-SDI countries on the map may therefore be an underestimation of the true burden of PCOS and may exaggerate the degree of inequality in PCOS prevalence between countries. In addition, a high growth trend in medium- and low-SDI regions may reflect the dual effect of a true increase in risk related to improved nutrition, urbanization, and increases in obesity rates, as well as an improvement in diagnostic capability.

The predominance of cases in women aged 20–39 years aligns with the condition’s peak expression during reproductive years. The highest burden was observed in middle-SDI regions, potentially attributable to rapid urban expansion, increased adoption of Western dietary and lifestyle patterns, and sociocultural shifts such as delayed childbearing and extended exposure to metabolic risk factors.^16,21,33–36^ By contrast, lower prevalence rates in low-SDI regions may reflect substantial underdiagnosis.^37^ In addition, as mentioned in the heterogeneity analysis, affected by the diagnostic criteria, low-SDI areas may have lower diagnosis and awareness of this condition and perhaps poor clinical databases that are reporting this condition.

Temporal patterns identified through the APC analysis indicated a post-2002 decline in high-SDI regions (rate ratio: 1.1→0.9), plausibly linked to the broader application of metformin and lifestyle-based interventions. In low-SDI regions, however, rate ratios increased from 0.8 to 1.0, suggesting limited access to such management strategies.^38^ Among later birth cohorts, particularly those born after 1990, the rate ratio in low-SDI regions surpassed that in high-SDI regions by approximately 0.25, suggesting elevated relative risks among younger cohorts in socioeconomically disadvantaged settings, likely implying additive effects from inherited epigenetic alterations and sustained androgenic exposures across generations.^39^

The health inequality metrics align with the observed patterns. The reduction in the CI: (0.24→0.10) suggests diminishing disparities associated with the SDI, potentially influenced by widespread global PCOS awareness initiatives. Nonetheless, sustained disparities in LMICs—where the ASPR remains 3.8 times higher than in high-income countries—signal the necessity for more precisely allocated resources. Divergent trajectories in China and India further illustrate this complexity; in China, the downward trend in inequality is attributed to broader insurance coverage for infertility treatments, whereas India’s static pattern reflects enduring structural impediments to rural health care delivery.

The frontier analysis revealed substantial deviations from the optimal prevalence–SDI trajectories in high-SDI countries such as the United Kingdom and the United States, likely reflecting the influence of obesogenic environments that offset advances in health care systems. In contrast, the proximity of Somalia and Niger to the efficiency frontier implies that a low SDI possibly does not inherently limit effective PCOS management when protective cultural factors—such as traditional dietary patterns—attenuate metabolic risks.^18,40^ These observations emphasize the need for tailored policy responses. In high-SDI settings, regulatory measures targeting environmental toxins and the integration of mental health services to address stress-associated insulin resistance warrant prioritization. In low- and middle-SDI contexts, increasing community-level screening *via* mobile health platforms and expanding access to subsidized pharmacological therapies, such as metformin, may enhance the disease management capacity. The notably flat slope of the leading fit line suggests a weak overall association between the ASPR and SDI in this setting. This diminished correlation may be attributable to opposing dynamics within SDI components; although enhanced health care infrastructure in high-SDI areas likely increases the diagnostic capture of PCOS, urbanization-associated environmental exposures may intensify clinical manifestations.

## Conclusions

Over the past three decades, a marked escalation in the global disease burden of PCOS has been observed, accompanied by substantial regional heterogeneity in prevalence. The disproportionately high rates among women aged 20–39 years highlight the importance of unified diagnostic standards and timely clinical interventions to mitigate long-term sequelae. Despite the overall progress in global health equity, persistent disparities within high-SDI regions necessitate targeted, context-adapted policy frameworks to enhance the efficiency and accessibility of care delivery.

## Limitations

Despite its broad analytical scope, this study presents several methodological constraints. First and foremost, our study relies on the GBD 2021 dataset, which defines PCOS solely based on the NIH criteria. This is a significant limitation, as the Rotterdam criteria are more commonly used in many clinical and research settings globally and capture a broader phenotype. This inherent discrepancy in case definition across different regions fundamentally challenges the comparability of prevalence estimates between countries. We acknowledge that the observed variations could be confounded by differences in local diagnostic practices and data sources (*e.g.,* some regions may primarily use NIH, others Rotterdam), rather than reflecting the true differences in the disease burden. Second, inconsistency in diagnostic criteria—such as differences between NIH and Rotterdam standards—complicates inter-regional comparability. The absence of phenotype-specific stratification, due to limitations in data resolution, further restricts interpretation; future research should incorporate analyses based on Rotterdam-defined subtypes. Third, the frontier analysis presumes a linear association between SDI and prevalence, which may inadequately represent the complex, multifactorial nature of the disease distribution. In addition, potential confounders, such as environmental exposures, were not systematically assessed, constraining the capacity to evaluate regional risk determinants. Finally, the dataset concludes in 2021, excluding potential post-pandemic shifts in health care utilization and lifestyle patterns. Mitigating these limitations will require standardized diagnostic criteria and strengthened data systems capable of supporting dynamic, region-specific surveillance of the PCOS burden.

## Data Availability

The data underlying this article are available in the Global Health Data Exchange at http://ghdx.healthdata.org/gbd-results-tool.

## Abbreviations Used

APC: age–period–cohort
ASPR: age-standardized prevalence rate
CI: concentration index
EAPC: estimated annual percentage change
GBD: Global Burden of Disease
LMICs: low- and middle-income countries
PCOS: polycystic ovary syndrome
SDI: socioeconomic development index
SII: slope index of inequality
UI: uncertainty interval
