# Supplementary material for: Global Trends in Polycystic Ovary Syndrome Burden, 1990–2021: Insights from the Global Burden of Disease Study
Source: Womens Health Rep (New Rochelle). 2025 Nov 11;6(1):1216–27. doi: 10.1177/26884844251395123 (PMC12670715; doi:10.1177/26884844251395123)
Supplement: Supplementary Tables [file 26884844251395123_supplementarytables.docx]

Supplementary Table 1. Prevalence counts in 1990 and 2021, the ASPR for PCOS by socio-demographic index (SDI)

| Location | **Prevalence(95%UI)**  **1990(**(Per 100,000)**)** | **2021(**(Per 100,000)**)** |
| --- | --- | --- |
| Global | 1372.77 (984.64to1891.60) | 1757.83 (1253.35to 2421.26) |
| High SDI | 3007.94 (2186.43to 4172.47) | 3554.30  (2624.21to4816.07) |
| Middle SDI | 1176.94 (825.22to1630.44) | 1971.07 (1395.79to2724.23) |
| Low-middle SDI | 745.13 (526.92to1040.82) | 1188.74 (828.69to1668.94) |
| Low SDI | 500.06 (353.56to711.57) | 714.31 (504.52to1007.40) |
| High-middle SDI | 1262.73 (892.37to1745.92) | 1817.62 (1277.35to2529.47) |

Supplementary Table 2. Prevalence counts in 1990 and 2021, the percentage change in prevalence and the case numbers for PCOS by socio-demographic index (SDI) and global regions.

|  | Prevalence (95% UI) | | | |
| --- | --- | --- | --- | --- |
|  | **1990** | **2021** | Percentage change in all ages, 1990-2021 (Per 100,000) | Percentage change in ASR, 1990-2021(Per 100,000) |
| Location | (Numbers) | (Numbers) |  |  |
| Global | 36651157.24 (26227943.17to50603929.78) | 69473252.37 (49531420to95724479.23) | 89.55% (85.03%to94.22%) | 28.05% (25.01%to31.08%) |
| High SDI | 13783058.46 (10021809.44to19223038.27) | 17573919.77 (12981150.82to23876520.56) | 27.5% (21.32%to36.14%) | 18.16% (12.65%to25.57%) |
| Middle SDI | 10580143.2 (7410905.26to14674450.81) | 24613369.91 (17452531.61to34074253.7) | 132.64% (123.22%to142.88%) | 67.47% (60.67%to75.4%) |
| Low-middle SDI | 4094311.31 (2868780.9to5765430.73) | 12118405.37 (8434180.98to17027257.26) | 195.98% (184.92%to209.49%) | 59.53% (53.24%to67%) |
| Low SDI | 1121930.97 (784807.02to1611458.42) | 3938251.29 (2745129.91to5589751.24) | 251.02% (236.82%to268.69%) | 42.84% (37.11%to49.86%) |
| High-middle SDI | 7047030 (4981606.26to9727416.64) | 11180894.89 (7874794.87to15590881.41) | 58.66% (53.33%to64.9%) | 43.94% (39.2%to48.86%) |
| Andean Latin America | 466365.78 (322493.99to653112.67) | 1172864.51 (808337.39to1649165.85) | 151.49% (129.79%to175.33%) | 37.77% (26.48%to50.81%) |
| Australasia | 444239.31 (326487.36to585953.23) | 701617.99 (500341.02to974912.97) | 57.94% (38.59%to81.58%) | 16.12% (2.07%to33.08%) |
| Caribbean | 222432.35 (149139.1to319229.69) | 360981.83 (245137.34to520033.49) | 62.29% (53.64%to71.43%) | 25.27% (18.92%to31.93%) |
| Central Asia | 118817.93 (79820.2to174949.99) | 237958.89 (163368.39to333464.66) | 100.27% (84.89%to113.88%) | 38.76% (28.69%to48.88%) |
| Central Europe | 116151.96 (76720.98to173921.49) | 119149.23 (82050.44to168514) | 2.58% (-8.58%to14.75%) | 23.2% (10.17%to37.56%) |
| Central Latin America | 2295521.99 (1578370.65to3199568.77) | 4072957.18 (2852240.56to5662452.4) | 77.43% (70.23%to84.75%) | 10.09% (6.11%to14.66%) |
| Central Sub-Saharan Africa | 110521.94 (76168.15to160819.7) | 444120.79 (305191.6to640542.02) | 301.84% (267.24%to341.66%) | 51.3% (38.86%to65.81%) |
| East Asia | 5607180.87 (3957250.24to7863221.6) | 10490358.54 (7423407.5to14808757.1) | 87.09% (75.09%to98.72%) | 83.12% (71.77%to94.74%) |
| Eastern Europe | 237422.15 (160523.33to339244.51) | 265678.69 (185374.43to381762.99) | 11.9% (6.13%to16.8%) | 28.32% (21.81%to34.12%) |
| Eastern Sub-Saharan Africa | 448071.03 (310256.93to646236.16) | 1438942.37 (1001296.83to2063339.49) | 221.14% (209.9%to235.3%) | 29.52% (24.79%to35.39%) |
| High-income Asia Pacific | 4402355.4 (3172199.05to6106156.59) | 4104982.68 (2922772.61to5775182.08) | -6.75% (-12.08%to-1.5%) | 10.26% (4.72%to16.33%) |
| High-income North America | 4469583.7 (3157929.56to6274672.88) | 6362238.32 (4742961.86to8324162.21) | 42.35% (25.45%to68.36%) | 25.35% (10.63%to47.82%) |
| North Africa and Middle East | 2463301.08 (1707181.32to3501813.36) | 6673431.49 (4672056.28to9434543.48) | 170.91% (157.81%to185%) | 33.99% (26.92%to40.74%) |
| Oceania | 40226.69 (27505.04to56891.57) | 124484.33 (86570.21to177516.43) | 209.46% (178.55%to236.12%) | 38.61% (25.27%to50.35%) |
| South Asia | 3294301.2 (2344772.01to4593824.79) | 11291117.08 (7950085.92to15832639.77) | 242.75% (222.38%to269.54%) | 76.71% (66.1%to90.73%) |
| Southeast Asia | 3682948.82 (2589198.76to5201509.94) | 10520027.69 (7378813.87to14809823.5) | 185.64% (166.63%to205.02%) | 86.73% (73.71%to100.3%) |
| Southern Latin America | 296321.47 (204840.66to431020.07) | 667604.83 (469021.54to956761.41) | 125.3% (107.55%to146.54%) | 59.59% (47.27%to74.35%) |
| Southern Sub-Saharan Africa | 232808.63 (160575.02to334631.38) | 480389.59 (328945.22to679589.28) | 106.35% (96.18%to116.82%) | 25.74% (20.03%to32.41%) |
| Tropical Latin America | 448844.15 (304409.01to647659.16) | 746471.68 (514676.97to1057462.85) | 66.31% (57.21%to75.3%) | 9.19% (4.2%to14.73%) |
| Western Europe | 6815142.46 (4796290.79to9455180.18) | 7455929.27 (5232259.44to10460556.82) | 9.4% (5.38%to13.39%) | 11.15% (7.21%to15%) |
| Western Sub-Saharan Africa | 438598.33 (307139.67to633877.78) | 1741945.38 (1209889.21to2493010.7) | 297.16% (279.57%to319.43%) | 43.97% (37.27%to52.39%) |
